# Genetic and Life Style Risk Factors for Recurrent Non-alcoholic Fatty Liver Disease Following Liver Transplantation

**DOI:** 10.3389/fnut.2021.787430

**Published:** 2022-01-14

**Authors:** Speranta Iacob, Susanne Beckebaum, Razvan Iacob, Cristian Gheorghe, Vito Cicinnati, Irinel Popescu, Liana Gheorghe

**Affiliations:** ^1^“Carol Davila” University of Medicine and Pharmacy, Bucharest, Romania; ^2^Center for Digestive Diseases and Liver Transplant, Fundeni Clinical Institute, Bucharest, Romania; ^3^Center of Excellence in Translational Medicine, Fundeni Clinical Institute, Bucharest, Romania; ^4^University Hospital Munster, Munster, Germany

**Keywords:** genetic, liver, NAFLD, NASH, liver transplant

## Abstract

Recurrent or *de novo* non-alcoholic fatty liver disease (NAFLD)/non-alcoholic steatohepatitis (NASH) following liver transplantation (LT) is a frequent event being increasingly recognized over the last decade, but the influence of recurrent NASH on graft and patient outcomes is not yet established. Taking into consideration the long term survival of liver transplanted patients and long term complications with associated morbidity and mortality, it is important to define and minimize risk factors for recurrent NAFLD/NASH. Metabolic syndrome, obesity, dyslipidemia, diabetes mellitus are life style risk factors that can be potentially modified by various interventions and thus, decrease the risk of recurrent NAFLD/NASH. On the other hand, genetic factors like recipient and/or donor PNPLA3, TM6SF2, GCKR, MBOAT7 or ADIPOQ gene polymorphisms proved to be risk factors for recurrent NASH. Personalized interventions to influence the different metabolic disorders occurring after LT in order to minimize the risks, as well as genetic screening of donors and recipients should be performed pre-LT in order to achieve diagnosis and treatment as early as possible.

## Introduction

Non-alcoholic fatty liver disease (NAFLD) is a highly prevalent condition in Western Europe and USA, but has also an increasing trend in Southern and Eastern European countries and Asia as stated by the HEPAHEALTH Project (1). It is now the most frequent chronic liver disease worldwide (25% of all adults) and represents a major global public health challenge (2) and a cause of significant morbidity and mortality. NAFLD is a liver disease comprising different variants (3) from steatosis (non-alcoholic fatty liver, NAFL), in which plethoric hepatic fat is shown, and non-alcoholic steatohepatitis (NASH), a necroinflammatory form of the disorder manifested by histological inflammation and hepatocyte ballooning that conducts to severe liver fibrosis with end stage liver disease (>20% in NASH patients) and hepatocellular carcinoma requiring liver transplantation (LT). NAFLD/NASH can be present in the patient awaiting LT, but also in the donors because of increased risk of cardiovascular events, the major cause of death in people with NAFLD (4).

Transplant candidates with NASH commonly have certain metabolic comorbidities supplementary to the complexity of managing the complications of chronic liver disease. Obesity escalates the risk of decompensation while on the waiting list and can represent a surgical technical challenge (5). Sarcopenic obesity is multifactorial, affects up to 35% of patients awaiting LT and is associated with increased morbidity and mortality compared to either disease alone, as well as worse survival after LT (6, 7). The overall prevalence of NAFLD and NASH among patients with type 2 diabetes mellitus (T2DM) is 55.5% and, respectively, 37.3% (8), thus T2DM being another factor implicated in prognosis of patients with NASH related cirrhosis awaiting LT and following LT.

Recurrent or *de novo* NAFLD/NASH following LT is a frequent event being increasingly recognized over the past decade (9). The influence of recurrent NASH on graft and patient outcomes is not yet clearly stated. Several data suggest that it does not impact graft and patient survival (10–12), but there is a large variation in the diagnostic modalities, protocol liver biopsies or non-invasive evaluation of fibrosis and follow-up intervals. However, there are publications analyzing the factors that influence the occurrence of NAFLD/NASH after LT and demonstrate the association with adverse post-LT outcomes related to liver and non-liver related events (13–17).

## Frequency, Progression and Significance of Post-transplant NAFLD

Recurrent and *de novo* NASH are increasingly being reported in post-LT NASH recipients, and quick diagnosis through non-invasive serological or imaging tests, followed by liver biopsy if needed, will help early intervention to avoid progression of NASH, and its related complications in the post-transplant period.

According to previous studies (14, 17, 18) there are variable prevalence of *de novo* NAFLD or recurrent NAFLD/NASH with different outcomes after LT. Recurrent NAFLD appears to be an earlier, more severe and with negative patient and graft outcomes. The recurrence of NAFLD has been reported to occur in 8.2–62.5% of recipients over variable follow-up periods ranging from <6 months to 10 years, and the rates for steatohepatitis have ranged from 4 to 33% over follow-up periods ranging from 6 weeks to 20 years. The rates of advanced fibrosis have ranged from 0 to 33% (short-term 6–12 months) or even 71.4% at 5 years after LT (14, 19, 20). One study even showed that almost 90% of patients developed recurrent NAFLD, but only 25% of them had advanced fibrosis following LT (21).

Taking into consideration the long term survival of liver transplanted patients and long term complications with associated morbidity and mortality, it is important to define and minimize risk factors for recurrent NAFLD/NASH.

## Life Style Risk Factors for Recurrent NAFLD After LT and Potential Interventions

Metabolic syndrome (MS) has been described in 43–58% of LT recipients. Obesity, dyslipidaemia, diabetes or insulin resistance, as well as certain immunosuppressive agents after LT are frequent predictors of recurrence of NAFLD after transplantation (22).

### Obesity

Obesity is encountered in more than one-third of all transplant recipients. Majority of the weight gain occurs during the first 1–3 years (23, 24), but persists to increase over the following years with an enlargement in abdominal girth and body fat content and corresponding low lean body mass. Obesity at 1-year post-transplantation shows a 2-fold increased mortality risk. Interventions to preclude the earliest weight gain might be more promising than later weight-loss endeavors.

Post-LT obesity management should comprise the same algorithm as in other obese persons: diet and exercise, pharmacologic therapy and surgical or endoscopic bariatric procedures. Weight loss is associated with improvement of recurrent NASH and better long term outcome. Weight loss medication can be used in this patient population, but the choice of medication should be individualized.

Orlistat, acting by directly stopping absorption of ~30% of dietary triglycerides, was evaluated in the post-LT setting and proved to be safe (25), but there are no data regarding its efficacy. Orlistat should be given at least 3 h before or after calcineurin inhibitors and levels should be monitored closely. There are no recorded interactions with antimetabolites or mammalian target of rapamycin (mTOR) inhibitors (26).

Liraglutide, a long-acting glucagon-like peptide-1 (GLP-1), appears to have no interactions with the immunosuppressive therapies and to have also cardio-protective effects in patients with known atherosclerotic disease or heart failure, making it an interesting option in these high risk patients. Following LT, liraglutide can be chosen in patients with diabetes mellitus, end stage renal disease or multiple drugs for different comorbidities, as well as in the early post-LT period to help avoid weight gain and possibly result in modest weight loss (27). Marked weight loss in patients with type 2 diabetes has also been noted in studies of semaglutide, a longer-acting GLP-1 analog, but there are no studies in LT recipients (28, 29).

Phentermine-topiramate suggests having the highest weight loss influence, by directly creating blockade of absorption of ~30% of dietary triglycerides. However, possible side effects are mentioned such as neuropsychiatric disorders, cardiovascular comorbidities, and drug-drug interactions that could limit their use. There are no known interactions with posttransplant immunosuppressants, but there are no data on the use of phentermine-topiramate following post-solid organ transplant setting (30).

Naltrexon-bupropion was authorized as a weight loss drug in 2014, leading also to improvement of fasting blood glucose and dyslipidemia. There are no data specific to the benefit of naltrexone-bupropion in the post-transplant setting, but there is no established interaction with post-transplant immunosuppressive medication. However, bupropion is a strong CYP2D6 inhibitor and can elevate the serum concentration of many drugs (26).

There is no specific immunosuppression strategy that has been shown to be useful in preventing weight gain after LT; however, immunosuppression should be tailored to diminish to minimum the risk of metabolic complications (30).

Bariatric surgery is also possible, may be safe and feasible after LT for weight loss, but may be more technically demanding, and is linked with elevated morbidity when compared with non-LT patients (17, 31). However, bariatric surgery should be taken into consideration for treatment of recurrent NAFLD because it ameliorates steatosis and steatohepatitis in most of the patients and improves or resolves liver fibrosis in 30% of patients (32).

Dyslipidemia occurs in 30–60% of LT recipients, being a major risk factor for allograft steatosis and posttransplant cardiovascular-related morbidity and mortality, and often continues despite dietary changes. A fasting lipid profile should be done every year in all LT recipients. mTOR inhibitors produce a stronger dyslipidemic effect compared to calcineurin inhibitors. Sirolimus proved to worsen hyperlipidemia in a dose-dependent manner (33).

Hypertriglyceridemia is the most common dyslipidemic change. Life-style changes should be realized when the low-density lipoprotein cholesterol (LDL-C) level is >100 mg/dL, although dietary modification alone is often inadequate, making pharmacotherapy necessary (24). Different circulating lipid components have varying effects. While circulating triglyceride (TG) levels are associated with the development of hepatic steatosis due to the imbalance between TG synthesis and breakdown process in hepatocytes, LDL-C is closely related to cardiovascular complications. Both lipid components should be addressed to reach different aspects of metabolic syndrome. The therapeutic goal for LDL-C should be below 100 mg/dL (even <70 mg/dL) in order to decrease the high cardiovascular risk in NASH patients after LT. Cholesterol is also a major lipotoxic molecule in NASH development. The gut microbiome represents an environmental factor contributing to the development of NAFLD and there are studies suggesting that dietary cholesterol caused advanced fibrosis by cholesterol-induced gut microbiota changes and metabolomic alterations (34). Thus, cholesterol inhibition and manipulation of the gut microbiota and its related metabolites might represent effective strategies in preventing NAFLD, but no studies are yet in the LT recipients.

Similar to non-transplant patients, statins are the drug of choice being usually well-accepted. Low doses of statins at beginning with slight increase as required and close follow-up should be taken into consideration. Pravastatin and fluvastatin are not metabolized by the CYP3A4 isoenzyme and should be first choice in post-LT recipients. Ezetimibe that acts through inhibition of enterohepatic recirculation of lipids, proved to effectively treat hypercholesterolemia with few side effects and to have no interaction with immunosuppressive agents. However, both pravastatin and fluvastatin are of low potency. Thus, combination therapy using ezetimibe will often be required to reach LDL-C targets. Alternatively, rosuvastatin is a substantially more potent option and is also not metabolized by cytochrome P450 (CYP) 3A4 (35).

Fibrates can be also safely used in patients with high triglyceridemia levels over 600 mg/dL, but caution is required when co-administrated with statins due to high risk of myotoxicity and renal dysfunction. For patients with hypertriglyceridemia, fish oil can be used with minimal side effects except potential increase of low-density lipoprotein level (18, 30).

### Diabetes Mellitus

One-third of LT recipients have type 2 diabetes mellitus (post-transplant diabetes mellitus, PTDM), requiring long-term therapy and follow-up. There is lot of evidence that people with T2DM are at high risk of developing NASH, but also that NAFLD may precede and/or develop T2DM, hypertension and atherosclerosis (36). This complex link between NAFLD and T2DM can be extrapolated to post-LT recipients. Treatment of NAFLD/NASH patients could avoid T2DM occurrence and/or progression, but, also the other way around.

Recipients with PTDM are handled just the same as patients with type 2 diabetes mellitus in the general population and the aim is to normalize target values and re-establish metabolic control. Dietary and lifestyle modification are of great importance, but are usually unsatisfactory in this population, with most patients requiring pharmacological therapy with oral agents or insulin.

Metformin and thiazolidinediones, influencing insulin resistance, proved to have benefit on biochemical and metabolic features of NAFLD, but amelioration of patients' histological response or fibrosis was modest and studies were usually short-term, thus liver-related long-term outcomes could not be evaluated (37).

Sodium–glucose cotransporter 2 inhibitors (SGLT2i) and glucagon-like peptide-1 receptor agonists (GLP-1RAs) are now accepted as the best therapeutic option for patients with T2DM and cardiovascular disease, heart failure and/or chronic kidney disease. These two types of drugs determine weight loss, making them an attractive option for patients with associated obesity, and offer promising effects in reducing liver fat content (37, 38). However, there are no clinical studies performed in post-LT NAFLD/NASH patients with these two drug classes although this therapeutic approach would be completely justified.

Bariatric surgery has recently proved to be one of the most effective therapeutic options for T2DM through weight-dependent and weight-independent mechanisms (39). Factors associated with diabetes remission consists of duration of diabetes prior to surgery <4 years, higher C-peptide, younger age and use of oral agents or diet to control diabetes (40).

Due to the increased prevalence of NAFLD worldwide, along with a reduced organ pool donation in many countries, usage of donor grafts with steatosis is now rather common. Donor graft steatosis is also a significant risk factor for post-LT recurrence of NASH (41). Defatting strategies, like pharmacological agents (e.g., forskolin, peroxisome proliferator-activated receptor (PPAR) -alpha ligand, hypericin, scoparone, PPAR-delta ligand, visfatin, L-carnitine) and hypothermic or normothermic machine perfusion have been shown to decrease hepatocyte steatosis (42). To achieve significant defatting, the protocol of choice should shift the balance toward more efficient TG breakdown (lipolysis) and excretion of related byproducts, as well as minimizing TG synthesis. There is still much research to be done on how best to modulate this lipid metabolism using cocktails of agents or *ex vivo* machine perfusions in order to achieve rapid defatting without adversely affecting viability and other critical liver functions. Short term survival and functionality of steatotic livers for which TG content has been dramatically reduced is already proven (43), however long term prevention of post-LT complications is not yet established.

## Genetic Risk Factors for Recurrent NAFLD After LT

There are few studies mentioning genetic influences on NAFLD recurrence post-LT. The range of recurrent NAFLD is wide and causes for this interindividual variability may be at least partially associated to differences in genetic background of both recipient and donor.

Finkenstedt et al. (44) showed that recipient patatin-like phospholipase domain containing 3 (PNPLA3) rs738409 was correlated with graft steatosis according to the 5-year post-LT computed tomography imaging. Kim et al. (45) found that the presence of the rs738409-G risk allele in both donor and recipient was an important risk factor for 1 year post-LT histologically proven NAFLD. Other data by Trunecka et al. (46) proved donor PNPLA3 rs738409 is a powerful risk factor of graft steatosis based on histologic findings on liver biopsy. The actual insight into the role of the p.I148M mutated PNPLA3 protein in liver fat turnover should favor the hypothesis that donor, but not recipient PNPLA3 genotype is critical for fat aggregation in the liver graft (47).

The donor TM6SF2 (transmembrane 6 superfamily member 2) c.499A allele is an independent risk factor of liver graft steatosis following LT in addition to the effects of donor PNPLA3 c.444G allele (48). The TM6SF2 p.E167K (c.499G>A) variant is important in patients with NAFLD, being associated with more severe steatosis, necroinflammation and advanced fibrosis/cirrhosis. Variants in the genes encoding glucokinase regulator (GCKR) and membrane bound O-acyl transferase 7 (MBOAT7) also contribute to the risk of NAFLD, by increasing *de novo* lipogenesis and altering the remodeling of phospholipid.

The study by John et al. (49) newly indicated that recipient adiponectin (ADIPOQ) rs1501299 and rs17300539 polymorphisms are associated with *de novo* NAFLD among patients transplanted for hepatitis C. *De novo* diabetes mellitus, as risk factor for post-LT NAFLD was associated with the following SNPs: recipient angiotensinogen (AGT) rs699; recipient mTOR rs2295080 (only following everolimus use); recipient ADIPOQ rs1501299 and rs822396; donor and recipient small ubiquitin like modifier 4 (SUMO4) rs237025 (50).

Our group recently demonstrated that the allele 1993C of the SNP rs4794067 of gene TBX21 (T-box transcription factor 21), but not CYP3A5^*^3 genotype may predispose to the development of late significant fibrosis and severe steatosis of the liver graft (51). The functional polymorphism TBX21-1993T/C (rs4794067) increases the transcriptional activity of the TBX21 gene (essential for Th1 polarization) resulting in a preponderance of a Th-2 or Th17 response.

Whenever genetic screening of recipients and donors identifies high risk genotypes for NASH, it is of paramount importance to control the modifiable risk factors and to intensify screening after LT for early detection of NAFLD/NASH.

**Figure 1:**
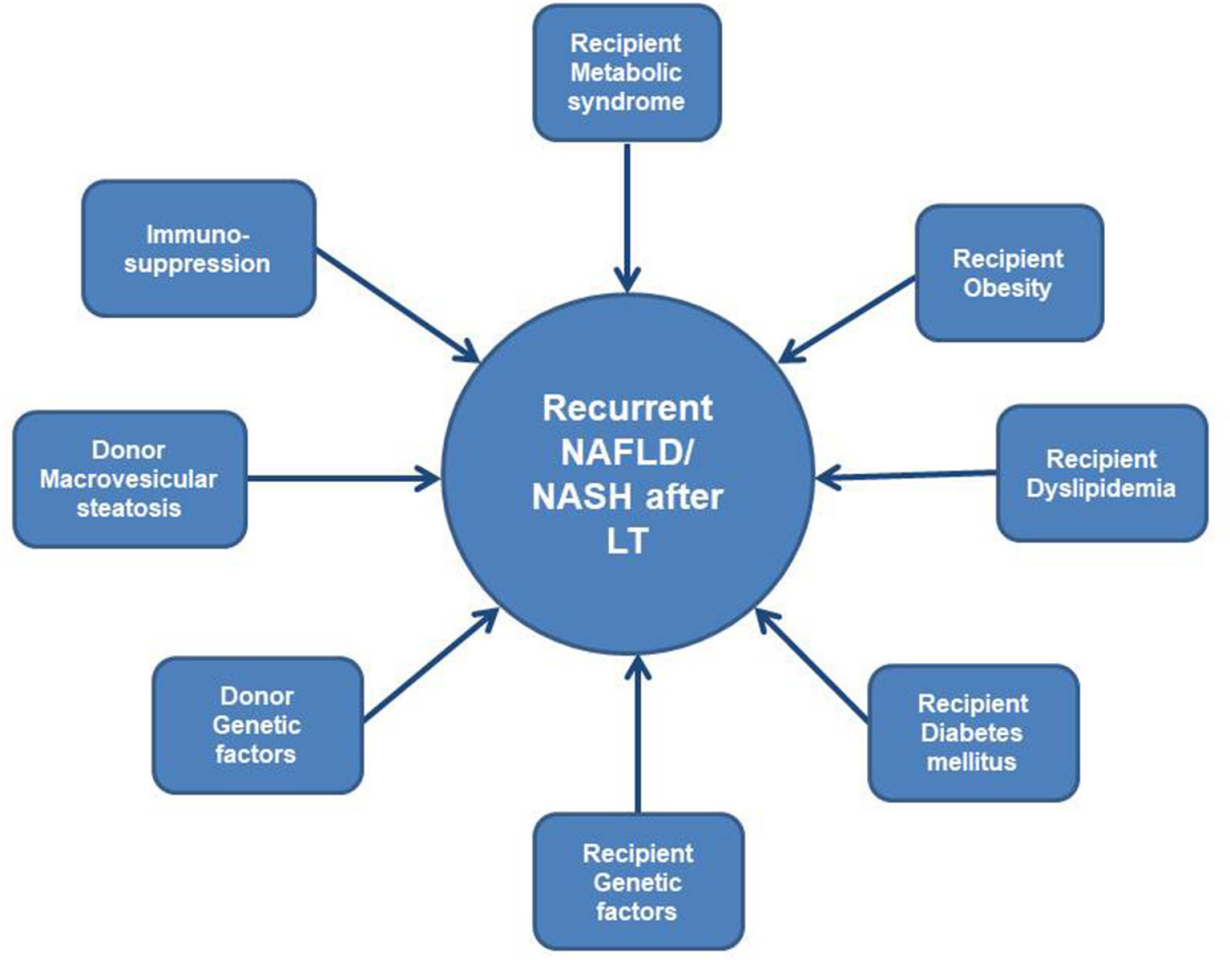
Modifiable and genetic risk factors for recurrent NAFLD/NASH after LT.

Screening for genetic risk factors before and after LT is very complex and interrelated (Figure 1). Multiple recipient and donor genetic factors are implicated in occurrence of all variants of NAFLD such as: risk factors for insulin resistance, for steatosis, for obesity and dyslipidemia, for metabolisation of immunosuppression, for gut microbiota, thus use of this data in clinical practice is still under investigation and constitutes one of the limitations of this review.

## Discussion

NASH remains the fastest growing indication for LT worldwide and recurrent NAFLD is common. There remains a need for long-term studies in this patient population to specifically address approach to diagnosis of recurrent NASH, preventive measures, treatment and implications.

Patients with histologically established posttransplant NASH have elevated risk of poor outcome as one third of them die within 5 years of the diagnosis and 26% develop a cardiovascular event. Almost one third of patients with recurrent NASH may develop bridging fibrosis/cirrhosis at 5 years after LT (17, 18).

Transient elastography (TE) is an ideal, non-invasive and accessible method for diagnosing the stage of hepatic fibrosis post-LT in both viral [hepatitis C virus (HCV) vs. non-HCV] patients (52, 53). Our group proved that LT recipients can very well be evaluated for steatosis and fibrosis by TE with CAP (controlled attenuation parameter) (54). Screening of NASH via TE and CAP should notify the clinicians and patients to this additional comorbidity and the greater possibility for complications related to insulin resistance. Patients who are at high risk of developing MS after LT should receive personalized interventions in order to minimize the risks, and should undergo routine surveillance in order to achieve an earlier diagnosis and treatment. The influence of immunosuppression on the development of MS and NAFLD after LT was extensively discussed in other papers (55, 56) and will not be in the focus of this review. Weight loss through diet, lifestyle modifications, pharmacological agents or bariatric surgery is linked with resolution of NASH and improvement in liver fibrosis, and should be implemented in overweight LT recipients, with an objective of 7–10% decrease in body weight (30). An early diagnosis of MS will restraint associated comorbidities, thereby reducing the risk of cardiovascular events. Strength of our review consists in establishing patients at risk of recurrence of NAFLD through genotypic and phenotypic characterization at transplant that will help to interfere by targeted strategies to prevent recurrence of NAFLD/NASH.

## Author Contributions

SI, SB, and LG: conceptualization. SI and RI: writing-original draft. CG, VC, SB, and IP: writing-review and editing. IP, SB, and LG: visualization. All authors contributed to the article and approved the submitted version.

## Conflict of Interest

The authors declare that the research was conducted in the absence of any commercial or financial relationships that could be construed as a potential conflict of interest.

## Publisher's Note

All claims expressed in this article are solely those of the authors and do not necessarily represent those of their affiliated organizations, or those of the publisher, the editors and the reviewers. Any product that may be evaluated in this article, or claim that may be made by its manufacturer, is not guaranteed or endorsed by the publisher.
